# Compression enhances invasive phenotype and matrix degradation of breast Cancer cells via Piezo1 activation

**DOI:** 10.1186/s12860-021-00401-6

**Published:** 2022-01-03

**Authors:** Mingzhi Luo, Grace Cai, Kenneth K. Y. Ho, Kang Wen, Zhaowen Tong, Linhong Deng, Allen P. Liu

**Affiliations:** 1 Institute of Biomedical Engineering and Health Sciences, Changzhou University, Changzhou, Jiangsu People’s Republic of China; 2 Department of Mechanical Engineering, University of Michigan, Ann Arbor, MI USA; 3 Applied Physics Program, University of Michigan, Ann Arbor, MI USA; 4 Present address: Center for Molecular Imaging, Department of Radiology, University of Michigan, Ann Arbor, MI USA; 5 Department of Biophysics, University of Michigan, Ann Arbor, MI USA; 6 Department of Biomedical Engineering, University of Michigan, Ann Arbor, MI USA; 7 Cellular and Molecular Biology Program, University of Michigan, Ann Arbor, MI USA

**Keywords:** Compression, Breast cancer cell, Invasion, Piezo1

## Abstract

**Background:**

Uncontrolled growth in solid breast cancer generates mechanical compression that may drive the cancer cells into a more invasive phenotype, but little is known about how such compression affects the key events and corresponding regulatory mechanisms associated with invasion of breast cancer cells including cellular behaviors and matrix degradation.

**Results:**

Here we show that compression enhanced invasion and matrix degradation of breast cancer cells. We also identified Piezo1 as the putative mechanosensitive cellular component that transmitted compression to not only enhance the invasive phenotype, but also induce calcium influx and downstream Src signaling. Furthermore, we demonstrated that Piezo1 was mainly localized in caveolae, and both Piezo1 expression and compression-enhanced invasive phenotype of the breast cancer cells were reduced when caveolar integrity was compromised by either knocking down caveolin1 expression or depleting cholesterol content.

**Conclusions:**

Taken together, our data indicate that mechanical compression activates Piezo1 channels to mediate enhanced breast cancer cell invasion, which involves both cellular events and matrix degradation. This may be a critical mechanotransduction pathway during breast cancer metastasis, and thus potentially a novel therapeutic target for the disease.

**Supplementary Information:**

The online version contains supplementary material available at 10.1186/s12860-021-00401-6.

## Background

Cancer invasion is a cumulative result of multiple processes including directed cell migration and extracellular matrix (ECM) degradation. While these processes are well known to be mediated by chemical factors, physical factors such as compression-induced mechanical forces have also been identified as essential regulators of these processes [1]. For example, an increase of compression inside a solid tumor is accompanied by enhanced cell proliferation [2]. Compression is also experienced by the cancer cells during migration through capillary and confined tissue microenvironments [3, 4]. Recent in vivo studies show that compression stimulates tumorigenic signaling in colon epithelial cells [5], and pressure release can indeed be used as a clinical strategy to enhance the efficiency of anti-tumor treatment [6]. Interestingly, it is demonstrated in vitro that compression directly alters cancer cell proliferation and migration, and thus drives them to be more invasive [7–9]. However, it is still unclear whether compression can be sensed by the cancer cells and transduced into cellular behaviors that promote matrix degradation and ultimately enhance the invasive phenotype of the cancer cells.

Considering that compression stretches cell membrane and thus increases membrane tension, it may as well alter the cellular behaviors of cancer cells through tension-mediated conformational changes of proteins and lipids in the membrane [10]. In particular, the increase of membrane tension can activate several stretch-activated ion channels (SACs) including Piezo and transient receptor potential (TRP) channels [11–14]. Comparing to TRP channels, Piezo channels are known to respond to membrane tension with more exquisite sensitivity [15, 16]. On the other hand, studies in vivo show that Piezo channels mediate a variety of compression-associated physiological activities such as touch perception [11] and blood pressure sensing [17], as well as pathological processes such as breast cancer development [18]. In the latter case, the role of Piezo channels is even substantiated by the fact that the survival time of the breast cancer patients is negatively related to the mRNA expression level of Piezo1 in the primary tumor [18]. Interestingly, it has been shown that the response of breast cancer cells to compression is dependent on Piezo but not TRP channels [19]. And upon activation of Piezo channels (Piezo1 in particular), the corresponding calcium influx evokes several downstream signaling pathways including Src and extracellular regulated protein kinase (ERK) which in turn affect the dynamics of actin-based protrusion structures such as invadopodia/invadosomes that degrade ECM proteins and thus promote invasion [20, 21]. These data indicate that Piezo1 may be essential for the compression-enhanced cancer invasion. However, whether and how Piezo1 channels mediate compression-enhanced invasive phenotype of cancer cells has not been examined.

So far it is thought in general that SAC functions at “membrane force foci” such as caveolae [22]. This is because caveolae are cholesterol-enriched flask-like membrane invaginations that may rapidly flatten and disassemble in response to an increase in membrane tension and thus provide proper platforms for harboring and gating SACs [23–27]. As for Piezo1, structural analysis has shown that there is a pocket sandwiched between Piezo1 repeat B and C, which provides a binding site as a means of interaction with lipids [14]. Despite such evidence of the structure for interaction between Piezo1 and lipid, where Piezo1 actually locates in the cell membrane is not well established and it remains unclear whether Piezo1 activity is indeed regulated by caveolae.

In this study, we hypothesized that Piezo1 channels mediate the compression-enhanced invasive phenotype of cancer cells. To test this hypothesis, we examined in vitro cultured human breast cancer cells for their ability to invade and degrade extracellular matrix in the presence or absence of externally loaded compression, together with corresponding changes in Piezo1 and calcium signaling. We found that the compression promoted an invasive phenotype in breast cancer cells, characterized by enhanced matrix degradation, actin protrusion formation, and calcium signal initiation. More importantly, the phenotypic changes in these cells appeared to be mediated by the compression-induced Piezo1 activation, which in turn was dependent on the caveolar integrity.

## Results

### Compression enhanced invasion of breast cancer cells dependent on Piezo1

**Fig. 1:**
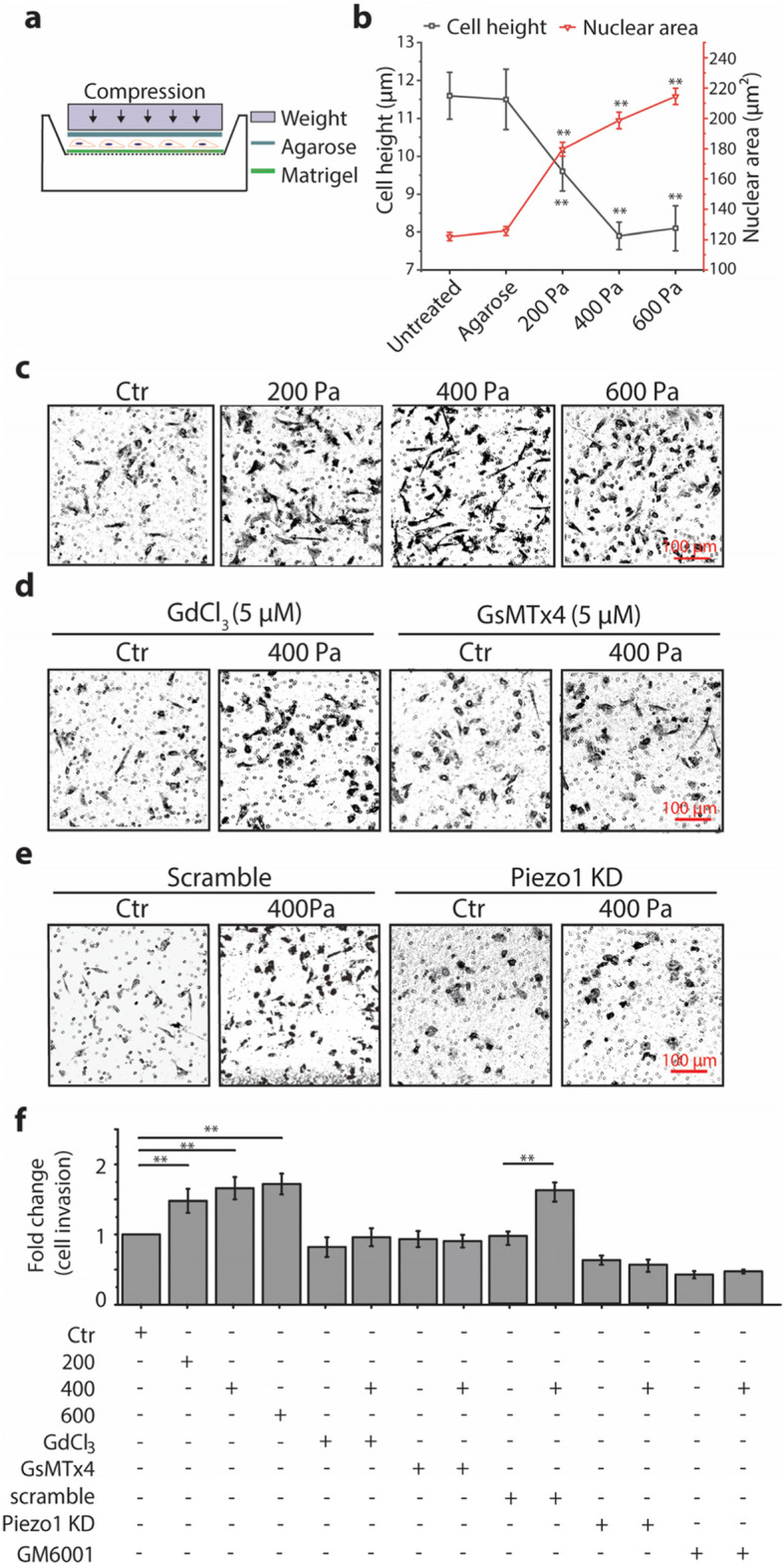
Compression enhanced invasion of MDA-MB-231 cells depending on Piezo1. Cell invasion was measured with in vitro transwell invasion assay. **a** Schematic diagram of the compression experiment using a transwell setup. Cells grown on a membrane filter (8 μm pore) coated with Matrigel for 6 h were covered with 1% of agarose gel and compressed with a specific weight. **b** The changes of cell height and nuclear area of MDA-MB-231 under compression. Data are presented as means ± s.e.m. *n* = 4, ^**^ *p* < 0.01 versus untreated groups. **c, d, e** Representative images of invaded cells stained with crystal violet under different compression and treated with gadolinium chloride (Gd^3+^), GsMTx4, or siRNA for Piezo1 under 400 Pa (Bar = 100 μm). **f** Quantification of the fold change of invaded cells. Data were presented as means ± s.e.m., *n* = 3, ^**^ *p* < 0.01 versus control (Ctr) groups

To test whether externally loaded compression enhances invasion of breast cancer cells, MDA-MB-231 cells were grown on a two-dimensional (2D) membrane filter (8 μm pore) coated with Matrigel and covered with 1% agarose gel and then compressed by a constant weight (Fig. 1a). The compression-induced stress levels in the experimental groups used in this study were 200, 400, and 600 Pa, which were considered pathophysiologically relevant as cells are reported to experience compressive stress at up to about 800 Pa in the core of solid breast tumor [9, 28]. To show whether compression squeezes the cell and cell nucleus, we first evaluated the height of the cancer cells by looking at side-view profiles of cells and the nuclear area of the cancer cells by looking at top-view profiles of the nucleus under compression. The results show that as the compression load increased, the cell height and the nuclear area significantly decreased and increased, respectively (Fig. 1b). It is worth noting that while the cell height ceased to further decrease from 400 to 600 Pa, the nuclear area kept increasing when the compression load increased. These data indicate that the compression indeed squeezed the cells and nuclei, which was most likely to alter the membrane tension, impact SACs activity, and thus change the invasion capacity of the cells [29]. As shown in Fig. 1c and f, more MDA-MB-231 cells had invaded through the Matrigel-coated transwell filters when exposed to the compression compared to their counterparts covered with 1% agarose only (control, Ctr). The results clearly show that the compression enhanced breast cancer cell invasion.

It has been reported that hypoxia enhances cancer cell invasion through the mediation of hypoxia-inducible factor (HIF)-1α [30]. In our experimental setup, it is possible that the weight on top of the cells might interfere with oxygen diffusion and cause hypoxia in the cells. Therefore, we treated the MDA-MB-231 cells with HIF-1α inhibitor (CAY10585, 10 μM) and then examined the cell invasion as described above. The results indicate that the compression-enhanced cancer cell invasion was largely unaffected no matter the cells were treated or not with HIF-1α inhibitor (Fig. S1). This suggests that the compression-enhanced cancer cell invasion was unlikely to involve hypoxia-related signaling, which is consistent with the hypothesis that the pores in the membrane may permit nutrient and oxygen diffusion to the cells in the event of physical confinement due to compression [9].

To test whether the compression-enhanced cancer cell invasion was mediated through SACs or more specifically through Piezo1, we pretreated the MDA-MB-231 cells with either Gd^3+^ (non-specific SACs inhibitor), or GsMTx4 (more specific Piezo1 inhibitor), followed by exposure to compression at 400 Pa. As shown in Fig. 1d and f, pretreatment with Gd^3+^ or GsMTx4 either partially attenuated or completely abrogated the compression-enhanced cancer cell invasion.

To further confirm the specificity of Piezo1 in mediating compression-enhanced cancer cell invasion, we examined the expression of Piezo1 in MDA-MB-231 cells. We found that Piezo1 was expressed in MDA-MB-231 cells in the form of punctate structures and located not only on the plasma membrane but also over the intracellular space and nucleus (Fig. S2a), which is consistent with data reported by Gudipaty et al. [31]. We then silenced the protein expression of Piezo1 in MDA-MB-231 cells by using siRNA. Western blot results confirmed that the efficiency of Piezo1 knockdown (KD) was ~ 70% (Fig. S2b). When the MDA-MB-231 cells with Piezo1 KD were exposed to compression at 400 Pa, the cells did not respond with enhanced cell invasion at all (Fig. 1e and f).

To test whether the compression-enhanced invasion was mediated by the function of matrix metalloproteinases (MMPs), we pretreated MDA-MB-231 cells with GM6001, a general MMP inhibitor, and then evaluated the invasion in the presence or absence of compression at 400 Pa. The results in Fig. 1e show that inhibition of MMP function with GM6001 completely abolished the enhancement of cell invasion in response to compression, suggesting that the compression-enhanced invasion capability of breast cancer cells was involved in the function of MMPs.

The same experiments carried out with 4 T1 cells (another breast cancer cell line) showed similar results as those with MDA-MB-231 cells (Fig. S3), confirming that the compression-enhanced breast cancer cell invasion and associated Piezo1 mediation were independent of the cell lines used.

### Compression enhanced matrix degradation dependent on Piezo1

Considering that cell invasion is a complex phenomenon involving cell proliferation, cell migration, and matrix degradation, it is necessary to examine each of these aspects for its role in the compression-enhanced invasion of breast cancer cells. We then measured cell proliferation and migration of MDA-MB-231 cells in the presence or absence of compression, respectively. The results show that compression increased cell proliferation, but the fold-change of compression-enhanced cell proliferation was always less than that of compression-enhanced cell invasion at the same load of compression as shown in Fig. S4 (i.e., 1.1 fold vs. 1.3 fold and 1.3 fold vs. 1.8 fold at 400 and 600 Pa, respectively). On the other hand, compression decreased cell migration as shown in Fig. S5. In addition, the compression-enhanced cell proliferation was attenuated when Piezo1 was knocked down in the cells (Piezo1 siRNA vs. scramble siRNA in Fig. S4). These data indicate that cell proliferation, but not cell migration, could contribute partially to the observed compression-enhanced invasion of the breast cancer cells.

**Fig. 2:**
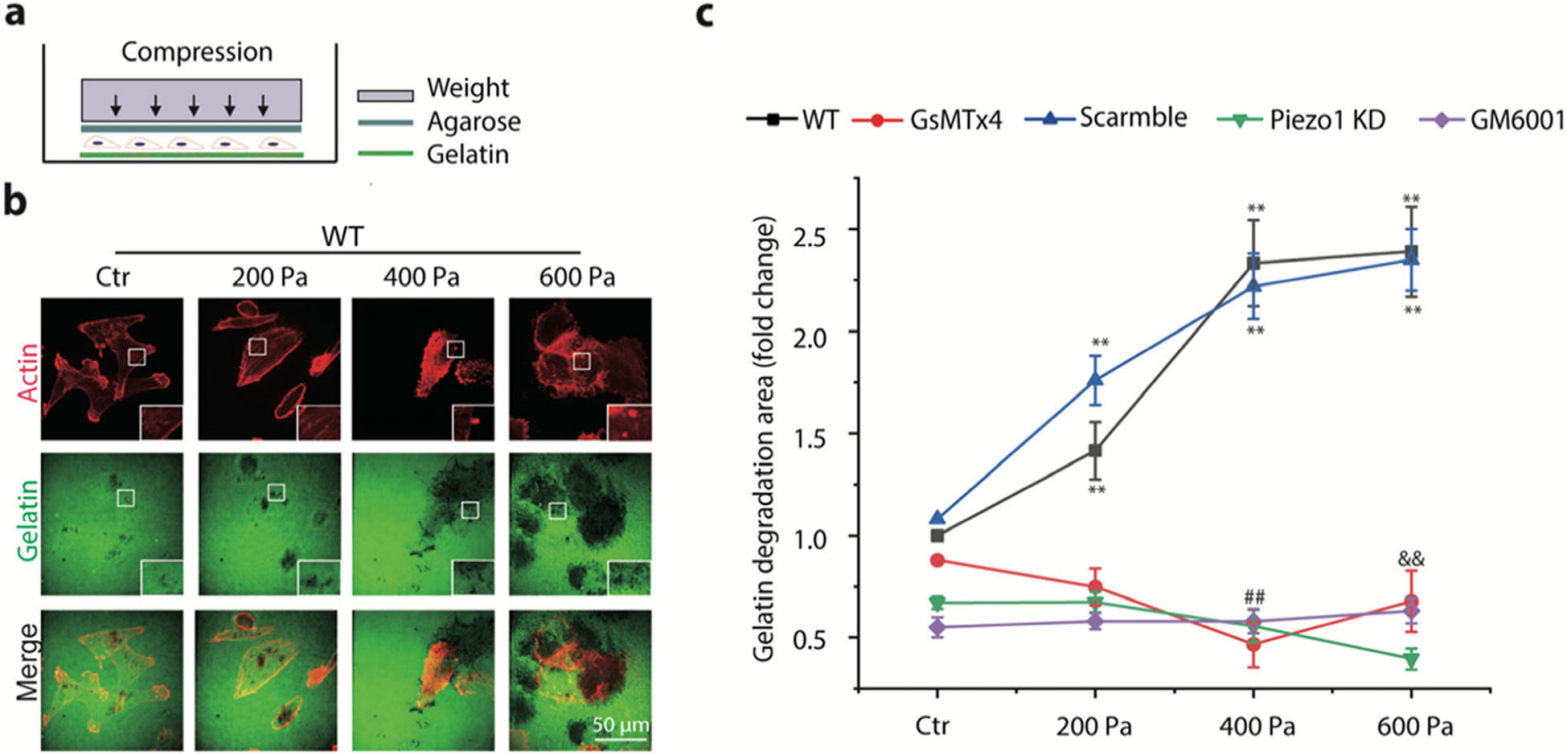
Compression promoted matrix degradation in MDA-MB-231 cells. **a** Schematic diagram of the experiment. Cells grown on a glass-bottom dish coated with FITC-conjugated gelatin for 8 h were covered with 1% of agarose gel and compressed with a specific weight. **b** Representative images (red: actin, green: gelatin) of compression-promoted gelatin degradation at the ventral side of the cell. Gelatin degradation was visualized by confocal microscopy (60X) as disappearance of green fluorescence. Inset images are magnified views of the boxed regions. **c** The fold change of gelatin degradation area under different treatment conditions (treated with GsMTx4, Piezo1 KD, or GM6001) as a function of compression normalized to gelatin degradation area at control (Ctr) groups; Data were presented as means ± s.e.m., *n* = 3, ^**^ *p* < 0.01 versus control groups, ## and $$ represent *p* < 0.01 versus 400 Pa and 600 Pa groups in wild type (WT), respectively

Since compression-enhanced invasion of the breast cancer cells was only partially due to cell proliferation and was involved in the function of MMPs, we suspect that compression may also influence cancer cells’ capability for matrix degradation. To investigate this, we examined the extent of matrix degradation of MDA-MB-231 cells seeded on FITC-conjugated gelatin-coated glass-bottom dish followed by application of compression (Fig. 2a). The fluorescence images showed dark puncta areas, corresponding to “holes” formed in the gelatin matrix due to degradation (Fig. 2b). Thus, we quantified the extent of matrix degradation, and the results showed that MDA-MB-231 cells exposed to compression from 200 Pa to 600 Pa exhibited a significant increase of gelatin matrix degradation as compared to their counterparts without compression (Ctr) (Fig. 2c). Similar to the case of cell invasion through Matrigel-coated transwell filters, pretreatment of MDA-MB-231 cells with GsMTx4 to inhibit Piezo1 or siRNA probe to silence Piezo1 expression completely abrogated the compression-enhanced gelatin matrix degradation in the cells (Fig. 2c). These data indicate that compression did enhance the matrix degradation capability of breast cancer cells in a Piezo1-dependent manner.

To test whether the compression-enhanced matrix degradation was mediated by MMPs, we pretreated MDA-MB-231 cells with MMP inhibitor GM6001, and then evaluated the matrix degradation in the presence or absence of compression as described above. The results in Fig. 2c show that inhibition of MMP function with GM6001 completely abolished the enhancement of matrix degradation in response to compression, suggesting that the compression-enhanced capability of breast cancer cells to degrade gelatin matrix was indeed mediated by MMP.

Furthermore, cancer cells are known to use actin protrusions known as invadopodia formed on the membrane to promote ECM degradation [20, 32–34]. Thus, we examined whether compression could promote invadopodia formation in MDA-MB-231 cells. We used immunofluorescence to visualize and identify invadopodia in MDA-MB-231 cells labeled with actin and cortactin, both of which are markers for invadopodia [35] (Fig. S6a). The number of invadopodia per cell was counted as actin-positive puncta and reported for MDA-MB-231 cells with or without pretreatment with siRNA probe to silence Piezo1, respectively, and with or without exposure to compression. The results show that compression increased the number of invadopodia per cell in MDA-MB-231 cells, which was significantly abrogated by silencing Piezo1 (Fig. S6b). These results demonstrate that breast cancer cells responded to compression with an increased number of invadopodia and thus promoted ECM degradation, which essentially depended on the activation of Piezo1.

### Piezo1 mediated compression-induced calcium signaling

**Fig. 3:**
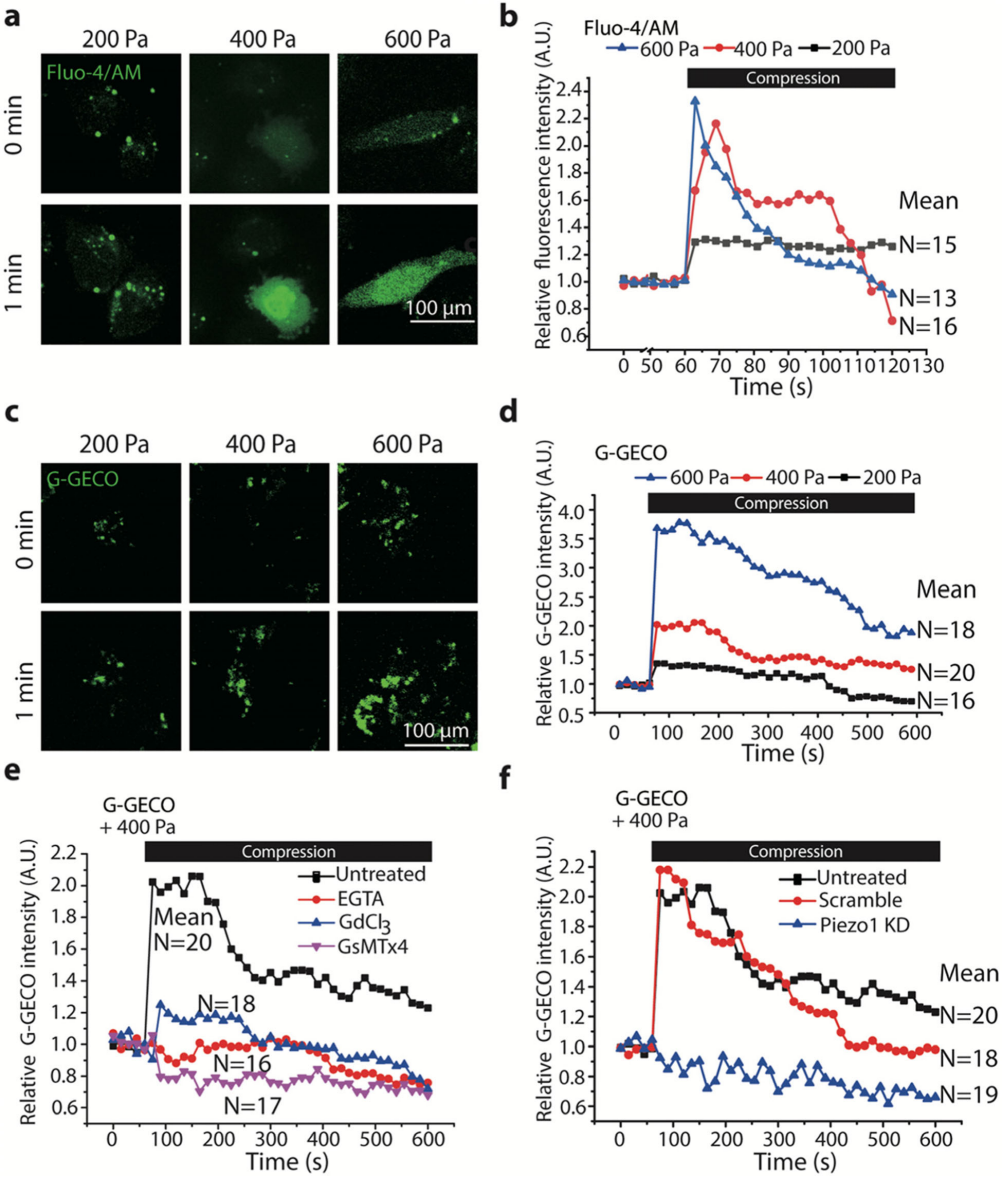
Compression induced calcium signaling in MDA-MB-231 cells. Representative images of intracellular [Ca^2+^] (**a** and **c**, bar = 100 μm) visualized by confocal microscopy (60X) and time-courses of changing relative mean fluorescence intensity (**b** and **d**) of Fluo-4 or G-GECO (normalized to time 0) in MDA-MB-231 cells labeled with Fluo-4/AM or transiently expressing G-GECO before (0 min) and after (1 min) exposure to compression at 200, 400, 600 Pa, respectively. **e, f** Time-courses of changing relative mean fluorescence intensity of G-GECO in MDA-MB-231 cells pretreated with or without EGTA, Gd^3+^, GsMTx4, and Piezo1 KD in response to 400 Pa compression. Each experiment assayed 10–20 cells and repeated three times. Black bars in **b**, **d**, **e**, **f** indicate the period of compression

To determine whether calcium signaling was involved in the compression-enhanced invasive phenotype of breast cancer cells, we labeled the cells with canonical calcium dye Fluo-4/AM or transiently transfected novel calcium biosensors green genetically encoded Ca^2+^-indicators for optical imaging (G-GECO) and performed live-cell imaging during application of compression to MDA-MB-231 cells. As shown in Fig. 3a, calcium signaling, as indicated by the fluorescence intensity of Fluo-4, was activated instantaneously upon exposure to compression (Supplementary video 1). The peak magnitude of activation (the relative fluorescence intensity of Fluo-4) increased from ~ 1.5 to ~ 2.5 fold as the compression increased from 200 Pa to 600 Pa (Fig. 3b). These results were confirmed by using G-GECO (Supplementary video 2, Fig. 3c and d). The peak magnitude of activation also increased from ~ 1.5 to ~ 3.5 fold as the compression increased from 200 Pa to 600 Pa.

The G-GECO system was used to measure the calcium signaling in the following experiments, because it is more convenient than the Fluo-4/AM system. We treated cells transfected with G-GECO with 2 mM ethylene glycol tetraacetic acid (EGTA) for 15 min to deplete extracellular calcium content before application of compression (400 Pa), which completely eliminated the compression-induced calcium signaling, suggesting the signaling was mainly due to influx of extracellular calcium (Fig. 3e). Furthermore, calcium influx induced by compression (400 Pa) was also abrogated when cells were pretreated with Gd^3+^ or GsMTx4 to block Piezo1 or siRNA probe to silence Piezo1 expression (Fig. 3f). Together, these observations support the finding that Piezo1 mediated the cellular response to compression via calcium influx.

### Caveolae regulated the location and function of Piezo1

**Fig. 4:**
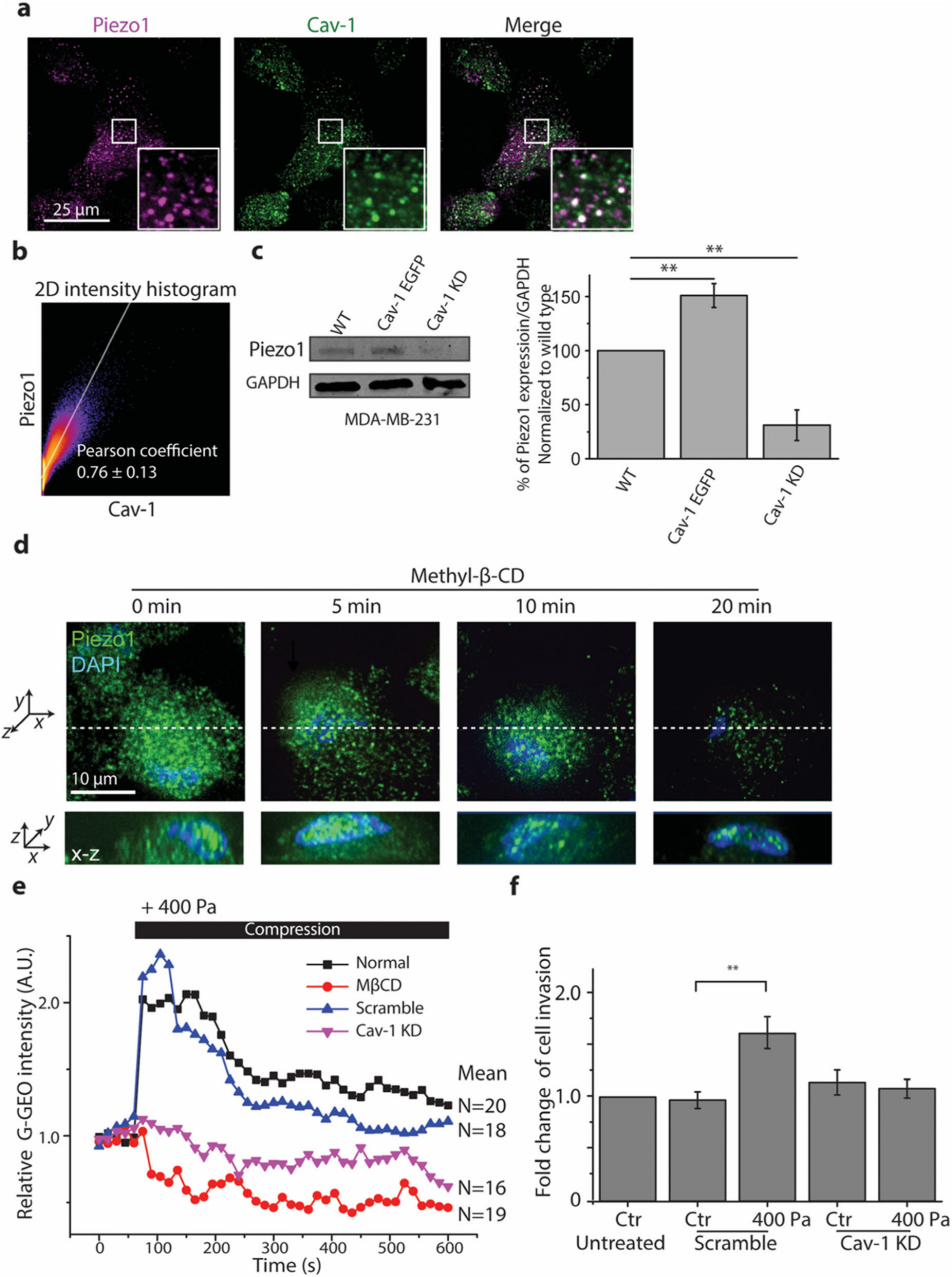
The expression and distribution of Piezo1 in MDA-MB-231 cells were regulated by caveolae. **a** Representative fluorescence images of Piezo1 (magenta) and caveolae (green) colocalization visualized by confocal microscopy (100X) and 2D intensity histogram output in MDA-MB-231 cells. Insets in both conditions show a magnified view of the boxed regions. **b** Representative image of 2D intensity histogram output of Coloc2 analysis performed using Fiji software. The text indicates the Pearson coefficient of the pixel-intensity correlation (*n* = 8). **c** Western blot images and quantification of Piezo1 expression in wild type (WT), Cav-1 EGFP expressing, and Cav-1 KD MDA-MB-231 cells (means ± s.e.m., *n* = 3). Cropped images of Western blots are shown and uncropped images are shown in Fig. S8b. ^**^ *p* < 0.01 versus WT groups. **d**, Representative fluorescence images of Piezo1 (green) and nucleus (blue) visualized by confocal microscopy (100X) after cells were treated with MβCD for 5 min, 10 min, and 20 min (upper panel: *x*-*y* view, lower panel: *x*-*z* view, white dashed line shows the position of a section of *x-z* view). **e** Time-courses of relative mean fluorescence intensity of G-GECO in MDA-MB-231 cells pretreated with or without MβCD, and Cav-1 KD in response to 400 Pa compression. Each experiment assayed 10–20 cells and repeated three times. The black bar indicates the period of compression. **f** Quantification of the fold change of invaded cells treated with siRNA for Cav-1 under 400 Pa. Data are presented as means ± s.e.m., n = 3, ^**^ *p* < 0.01 versus Ctr groups

Previous work suggests that cholesterol content that directly influences the formation of caveolae might regulate Piezo1 functions [36–39]. To test whether Piezo1 is located in caveolae, we first examined the distribution relationship between Piezo1 and caveolae. We found that both Piezo1 and caveolae (Cav-1) formed puncta structures and many of them were colocalized (Fig. 4a). The coefficient of colocalization in wild type cells (WT) was analyzed with Coloc2 procedure in Fiji software as shown in Fig. 4b. The results show that the classical Pearson coefficient was 0.76 ± 0.13, which indicates that Piezo1 and Cav-1 were highly colocalized. To test whether caveolae regulate the Piezo1 expression, we quantified Piezo1 protein expression level in MDA-MB-231 cells that were either wild type (WT), or transiently transfected with Cav-1 enhanced green fluorescent protein (Cav-1 EGFP), or siRNA probe for silencing Cav-1 expression (Cav-1 KD). We found that as compared to WT, Piezo1 expression was increased in Cav-1 EGFP cells while decreased in Cav-1 KD cells (Fig. 4c).

To verify the role of caveolae in regulating the Piezo location in the cell membrane, MDA-MB-231 cells were treated with 5 mM of methyl-β-cyclodextrin (MβCD) that dramatically reduced the number of caveolae (Fig. S7a). Consequently, the fluorescence intensity of Piezo1 appeared to decrease in the cell membrane, but increased in the nucleus at 5 min and up to 20 min (Fig. 4d), suggesting that caveolae regulated Piezo1 location in MDA-MB-231 cells.

To test the role of caveolae in regulating the Piezo1 function during compression, MDA-MB-231 cells were pretreated with either 5 mM MβCD or siRNA probe for silencing Cav-1 expression by about 60% (Fig. S7b) and then exposed to compression at 400 Pa. We found that compression-induced calcium influx was blocked in both the MβCD-treated and Cav-1 KD cells (Fig. 4e). Consistent with these results, Cav-1 KD also abrogated the compression-enhanced cancer cell invasion (Fig. 4f). These data indicate that at least in MDA-MB-231 cells the function of Piezo1 is dependent on caveolae.

### Piezo1 mediated compression-enhanced Src/ERK activation

**Fig. 5:**
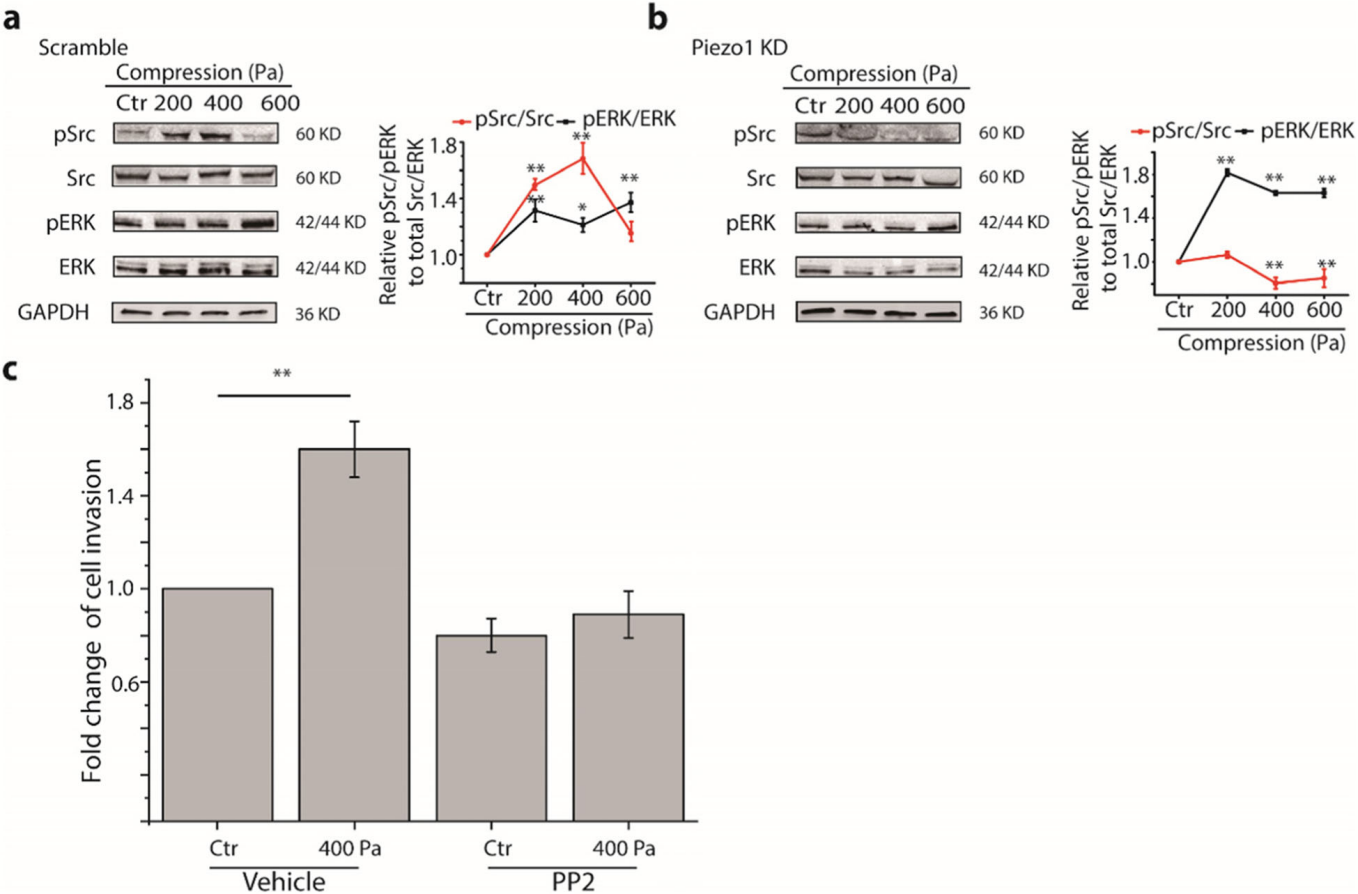
Compression enhanced the activity of Src and ERK. **a** Western blot analyses of the phosphorylation of Src and ERK in MDA-MB-231 cells pretreated with scramble probes in the absence or presence of compression at 200, 400, 600 Pa. Cropped images of Western blot are shown and uncropped images are shown in Fig. S8d-g. **b** Western blot analyses of the phosphorylation of Src and ERK in MDA-MB-231 cells pretreated with siRNA for Piezo1 in the absence or presence of compression at 200, 400, 600 Pa. Cropped images of Western blot are shown and uncropped images are shown in Fig. S8h-k. Relative phosphorylation levels were obtained by normalizing to GAPDH expression and value in control (Ctr) groups, *n* = 3. ^*^p < 0.05 versus Ctr groups; ^**^ *p* < 0.01 versus Ctr groups. **c** Quantification of fold change of invaded cells in 400 Pa compression to the Ctr group pretreated with DMSO (vehicle). Data are presented as means ± s.e.m., n = 3, ^**^ *p* < 0.01 versus Ctr groups

During invadopodia formation and maturation to degrade matrix, several signaling pathways are involved including Src/ERK pathways [21]. To test whether these signaling pathways are activated by compression, we quantified the phosphorylation of Src and ERK in MDA-MB-231 cells following compression. We found that compression significantly activated Src and ERK (Fig. 5a). Additionally, Piezo1 KD effectively abolished the compression-promoted signaling of Src, but not ERK (Fig. 5b), suggesting that Src, but not ERK was activated by compression in a Piezo1-dependent manner. We also treated MDA-MB-231 cells with either Src inhibitor PP2 or a blank vehicle, and found that compression-induced cell invasion was blocked in cells treated with PP2 whereas those treated with a vehicle increased cell invasion by ~ 1.6 fold at 400 Pa compression (Fig. 5c). This suggests that the compression-enhanced invasion of MDA-MB-231 cells was indeed mediated by Piezo1-dependent Src signaling.

## Discussion

In the present study, we first observed that in breast cancer cells, compression enhanced cancer cell invasion by promoting not only cell proliferation but also matrix degradation through the formation of actin protrusion. Additionally, we identified that Piezo1 mediated these processes and the invasive phenotype of the breast cancer cells also depended on the integrity of caveolae in the cell membrane. These findings provide the first demonstration that compression can enhance matrix degradation by breast cancer cells and Piezo1 is an essential sensor and transducer for such mechanical stress in breast cancer cells.

Invasion of cancer cells through ECM is a critical activity during cancer metastasis. Previous studies have shown that uncontrolled cancer growth can induce remarkable compression and thus trigger invasive phenotype in cancer’s leader cells, and the cancer cell invasiveness is directly related to the cell’s ability to form invadopodia [9, 32]. It is, however, unknown whether such compressive stress would affect the capability of cancer cells to induce ECM degradation. Here we report that, in consistency with the enhanced invasion of breast cancer cells, compression enhanced matrix degradation via promoting actin protrusions in the ventral sides of breast cancer cells. Thus, it is plausible that compression in the solid tumor might initiate invasion by enhancing the cancer cells’ capability of matrix degradation via actin protrusions. If that is the case in vivo, compression might promote cancer cells to ‘dig more holes’ in the basement membrane which provides a way for their metastasis.

While it is known that compression affects cancer progression, how cancer cells sense and respond to compression is not completely understood. Under compression, the cell membrane is likely to be stretched which in turn increases the tension and thus stimulates the stretch-activated channels (SACs) in the membrane. In this study, we found that compression indeed squeezed the cells, and induced a series of cell responses that were dependent on the activation of Piezo1. Piezo1 belongs to the family of Piezo channels that are the most notable SACs in mammalian cells gated by membrane tension [40]. It has been found that Piezo1 channels play essential roles in diverse physiological and pathological processes including cell migration [41, 42], and the Piezo1 mRNA expression level is highly correlated with the survival time of breast cancer patients [18]. Our study confirmed that Piezo1 channels are also essential in mediating the compression-enhanced invasion of breast cancer cells. We also found that both Piezo1 KD and Cav-1 KD significantly affected all aspects related to compression-enhanced invasion of MDA-MB-231 cells including cell proliferation and matrix degradation, but the cells seemed to be more sensitive to Piezo 1 KD than Cav-1 KD in their responses to compression. This perhaps is reasonable because even in the absence of weight-loaded compression, Piezo1 channels in the cells may have a basal activity due to the constant existence of atmospheric pressure and culture medium on top of the cells.

Emerging evidence indicates that caveolae harbor and modulate ion channels. For example, removal of caveolae via cholesterol depletion can disrupt the expression and distribution of TRPV1 channels on the plasma membrane [43]. Similarly, we found that depletion of cholesterol in MDA-MB-231 cells with MβCD caused Piezo1 to shift its localization from cell membrane to the nucleus. Interestingly, in stretch-triggered mitosis, Piezo1 was also observed to localize to the nuclear envelope [31]. Thus, it may be a general strategy for cells to regulate force-sensing through a functional relationship between caveolae and Piezo1. As our data suggested, caveolae might concentrate Piezo1 as the “mechanical force foci” which facilitates force sensing and transduction in mammalian cells.

Nonetheless, the mechanisms of how Piezo1 channels are gated by mechanical stress are still unclear. It has been reported that Piezo1 channels appear to be gated by the tension in the bilayer membrane according to the “force-from-lipid” principle, which is an evolutionarily conserved gating mechanism [44]. According to this paradigm, the activity and sensitivity of Piezo1 channels can be regulated by the lipid membrane because the physical properties of lipid membrane such as thickness, stiffness, and lateral pressure profile found within caveolae may be different from those of the surrounding membrane. In this context, it is plausible that cholesterol-enriched caveolae might affect the sensitivity of transmembrane channels such as Piezo1 via controlling the membrane pressure profile. For instance, disruption of caveolae by cholesterol depletion has been demonstrated to change membrane stiffness, and result in suppression of epithelial sodium and TRP channels [36, 38, 45]. Stomatin-like protein-3 has been reported to tune the sensitivity of Piezo1 channels by controlling the membrane mechanical properties through recruiting cholesterol [36, 46]. In this study, we found that the function of Piezo1 in compression sensing was regulated by caveolae. Taken together, it is likely that Piezo1 is located in the microdomain of cholesterol-rich caveola and is thus regulated by the caveolar integrity in order to function.

In addition, cells can also reorganize their cytoskeletal structures to adapt to the changing mechanical microenvironment. Among them, stress fibers are the essential cytoskeletal structures that control various cellular behaviors. Reports have shown that mechanical tension induces the assembly of stress fibers [47]. In this study, we found that cells under compression quickly assembled new stress fibers within 10 min (data not shown). This may be a requirement for the cells to quickly increase their mechanical strength in order to balance the compression.

Our results also demonstrate that some of the key pathways involved in mechanotransduction played important roles in regulating the compression-enhanced cancer cell invasive phenotype, including Src and calcium that are also linked to the formation and function of actin protrusions such as invadopodia [48, 49]. However, it remains unclear in detail how compression actually activates Piezo1 and then triggers calcium signaling. For example, it is still in question whether Piezo1 is activated by compression directly or indirectly via compression-derived stretch. Additionally, in this study, we mainly focused on the function of Piezo1 located in the plasma membrane, although it has been reported that Piezo1 can also locate in the intracellular compartments such as endoplasmic reticulum (ER) [50]. Since intracellular calcium signaling can arise from both influx of extracellular calcium into the cell through ion channels in the membrane and release of intracellular calcium stored in subcellular compartments such as ER, our results therefore could not completely exclude the possible role of intracellular calcium storage in mediating the compression-induced cellular responses.

In fact, there is increasing evidence that cells are able to transmit external mechanical forces to different organelles deep within the cell such as ER, where a number of mechanosensitive ion channels such as TRP and Piezo may be localized and activated correspondingly [51]. It is also reported recently that calcium release from intracellular calcium stores in response to a mechanical stimulus such as fluid shear stress was mediated by IP_3_ and ryanodine receptors, which are also vitally important in mechanotransduction [52, 53]. The potential roles of these factors in the compression-enhanced invasion of breast cancer cells are important open questions to be studied in the future.

For the effect of mechanical compression on cell proliferation of solid tumors, there are still many conflicting views. Some studies report that compression inside solid tumors inhibited cell proliferation and cell cycle transition [54, 55]. For example, Delarue et al. [55] reported that compression induced the blocking of cell cycle at the late of G1 checkpoint. On the contrary, Basson et al. [56] reported that enhanced extracellular compression promoted cell proliferation in several kinds of solid tumors including SW620, Caco-2, and CT-26 colon, MCF-7 breast, and MLL and PC3 prostate. These discrepancies may be due to the different experimental systems such as cell types, compression devices, and thus differential cellular response mechanisms [57]. In our experimental conditions, the compression seemed to promote proliferation of the cancer cells. Although this might have contributed to the results of compression-induced invasion of cancer cells, such contribution should be relatively small and insufficient to change the overall role of compression in promoting cancer cell invasiveness.

Finally, it is worthy to note that, in this study, we only evaluated the effect of uniaxial compression on breast cancer cells in a 2D culture model. However, cells in vivo grow and live in a 3D microenvironment, which may impact the force direction and change the dynamic response of the cells to compression. Additionally, we only assayed cell invasion in response to compression. Many other features of breast cancer cells such as the loss of acini morphologies in response to compression still need to be explored. For instance, Ricca et al. have shown that brief compression to a single malignant breast cancer cell in laminin-rich ECM can stimulate the formation of acinar-like structures, indicating that compression may cause malignancy reversion in breast cancer cells [58]. Furthermore, in this study we only investigated Piezo1 for its role in mediating the breast cancer cell response to compression. However, it has been reported that these cells also express Piezo2 for promoting mechanotransduction via RhoA activation and F-actin remodeling, raising the question of potential implications of other members of the Piezo family in the compression-induced cellular responses [59]. Therefore, further studies are required to fully elucidate the behaviors and associated underlying mechanisms of breast cancer cells in response to compression during tumor growth and metastasis.

**Fig. 6:**
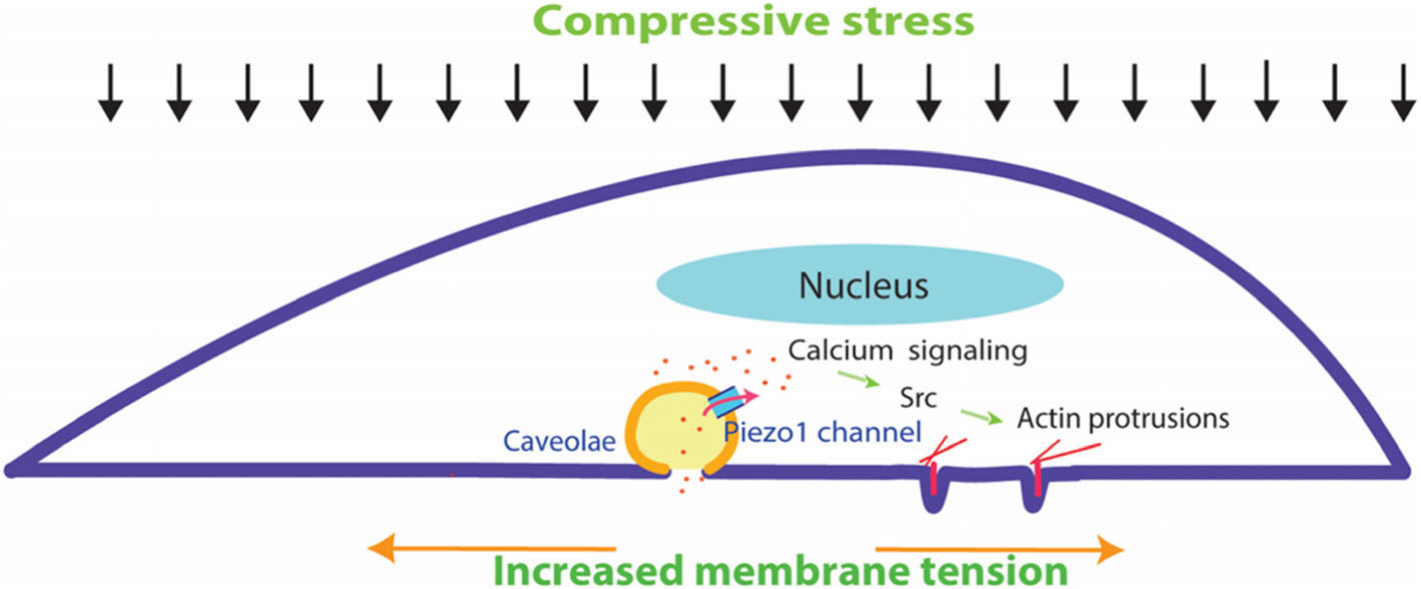
Model of compression-promoted invasive phenotype of MDA-MB-231 cells and associated signaling pathways. Together, vertical mechanical compression might increase the lateral plasma membrane tension and activate Piezo1 channels. The opening of Piezo1 mediates the influx of calcium and evokes the downstream signaling pathways such as Src. These activated signaling molecules promote actin protrusions at the ventral side of cells, which in turn mediate enhanced matrix degradation and cell invasion

In conclusion, our study provides a comprehensive understanding of the disparate systems involved in the context of compression-enhanced breast cancer cell invasion (Fig. 6), which may have relevance to the metastasis of malignant human solid tumors such as the breast cancer in vivo. Specifically, in a solid tumor the cancer cells may experience high compression due to uncontrolled proliferation and stiff ECM confinement, and such a mechanical microenvironment may ultimately facilitate compression-enhanced tumor cell invasion via matrix degradation. In this process, Piezo1 plays a crucial role in regulation of all the cellular behaviors associated with compression-enhanced invasion including cell proliferation, matrix degradation, cytoskeleton remodeling and intracellular Src and calcium signaling. These findings underscore the cardinal role of Piezo1 channels in regulating cancer cell invasion, and may inspire further development of anti-cancer drugs that use Piezo1 as a potential therapeutic target.

## Materials and methods

### Cell culture and preparation

All cell lines described below were purchased and used for the study. MDA-MB-231 cells (ATCC HTB-26), an invasive human breast adenocarcinoma cell line, were cultured in Dulbecco’s modified Eagle medium (DMEM) with 2 mM L-glutamine (# 11965–092, Thermo Fisher, Waltham, MA) supplemented with 10% fetal bovine serum (FBS, # 35–010-CV, Thermo Fisher), 100 units/mL penicillin, 100 μg/mL streptomycin, 2.5 μg/mL fungizone, and 5 μg/mL gentamicin (# 15750–060, Invitrogen, Carlsbad, CA) at 5% CO_2_ and 37 °C. 4 T1 cells (ATCC CRL-2539, a mouse breast cancer cell line) purchased from BeNa Culture Collection Corporation (# BNCC273810, Beijing, China) were cultured in RPMI-1640 (Gibco-Invitrogen, Carlsbad, CA) supplemented with 100 units/mL penicillin, 100 μg/mL streptomycin, and 10% FBS. For matrix degradation and invadopodia experiments, cells were incubated in invadopodia medium containing DMEM supplement with 5% Nu-Serum (# 355104, Corning, NY), 10% FBS, and 20 ng/mL EGF.

For labeling actin in live cells, stable cell lines expressing Lifeact-RFP were generated via lentiviral transfection. The lentiviral transfer plasmids pLVX-puro-GFP-Lifeact and pLVX-puro-RFP-Lifeact were cloned from RFP-Lifeact plasmid obtained from Dr. Gaudenz Danuser (UT-Southwestern). Briefly, lentiviruses were produced by transfecting human embryonic kidney 293 T cells (ATCC CRL-3216) with psPAX2 and pMD2.G (Addgene) and pLVX-puro-GFP-Lifeact viral vectors. Conditioned medium containing viruses were collected after 5 days and then used immediately to infect cells or stored at − 80 °C. Transduced target cells were selected with puromycin for 72 h.

For optical imaging of dynamic calcium signaling and caveolae localization in live cells, cell lines transiently expressing G-GECO (a green fluorescent genetically encoded calcium indicator) and caveolin-1 (Cav-1)-EGFP respectively were generated via plasmid transfection. The plasmids expressing G-GECO were a generous gift from Takanari Inoue (Johns Hopkins University) [60], and those expressing Cav-1-EGFP were from Ari Helenius (ETH Zurich). Briefly, cells were transfected with Lipofectamine-2000 (# 11668–019, Life Technologies, Carlsbad, CA). For 35 mm glass-bottom dishes, 6 μg plasmid DNA in OptiMEM transfection medium (# 31985062, Gibco, Waltham, MA) was used for each transfection. After 24 h at 37 °C, the transfection medium was replaced with a complete medium, and cells were processed 24–48 h later.

### Drug treatments

For experiments involving inhibitors, cells were exposed to the inhibitor for 0.5 h, unless stated otherwise, in the presence or absence of compression. For inhibiting the function of mechanically sensitive ion channels, cells were treated with gadolinium chloride (Gd^3+^, 5 μM, # 203289, Sigma) or GsMTx4 (5 μM, #ab141871; Abcam, Cambridge, MA). To remove calcium ions from the DMEM, EGTA (2 mM, # E3889; Sigma) was added to the medium. To disrupt caveolae in the membrane, cells were treated with 5 mM of methyl-β-cyclodextrin (MβCD, # SLBP3372V, Sigma). To evaluate the impact of HIF-1a, cells were treated with inhibitor CAY10585 (10 μM, # ab144422, Abcam). For inhibiting the activity of Src, cells were treated with PP2 (10 μM, Calbiotech, Spring Valley, CA). For inhibiting the activity of MMP, cells were treated with GM-6001 (a broad-spectrum MMP inhibitor, 15 μM, #CC1000; Sigma).

### Antibodies for immunofluorescence and Western blot

Antibodies used in immunofluorescence and Western blot include: anti-Tks5 polyclonal antibody (# 09–403-MI) and anti-GAPDH mouse monoclonal antibody (# CB1001) purchased from EMD Millipore (Billerica, MA); anti-Src rabbit antibody (# 2108), anti-p-Src (Y416) rabbit antibody (# 2101), anti-p44/42 MAPK (ERK1/2) mouse monoclonal antibody (# 4696), and anti-p-ERK1/2 (Thr202Tyr204) rabbit monoclonal antibody (# 4370) obtained from Cell Signaling Technology (Danvers, MA), respectively; anti-cortactin rabbit monoclonal antibody (# Ab81208) purchased from Abcam; anti-Piezo1 rabbit polyclonal antibody (# PA5–72974) and anti-Cav-1 rabbit polyclonal antibody (# PA1–064) obtained from Thermo Fisher.

### In vitro compression device

To investigate the effect of compression on cell behaviors, we used a previously described setup [9, 61]. Briefly, cells were grown either in a 35 mm culture dish with a glass bottom (# 12–565-90, Thermo Fisher, Waltham, MA) that was coated with/without gelatin, or in a transwell chamber with a permeable membrane of 8-μm pores that were coated with Matrigel. Then the cells were covered with a 1% soft agarose disk layer, and subsequently, a piston of specific weight was placed on top of the agarose disk to apply given compression to the cells underneath indirectly. The cross-sectional area of the piston (24 mm diameter) was 4.52 cm^2^ but its weight was variable at 9.22 g, 18.45 g, and 27.67 g, corresponding to a stress of 200 Pa, 400 Pa, and 600 Pa, respectively, on the cells. Cells prepared as such but not subjected to piston weight were used as control (Ctr). It needs to note that even cells in the control groups were also exposed to 1% agarose, a constant atmosphere pressure, and culture medium.

### RNA interference

To silence the expression of Piezo1 and Cav-1, Negative Control Medium GC Duplex #2 and siRNA interference for Piezo1 (# AM16708, Assay ID:138387, Thermo Fisher) and Cav-1 (# AM16708, Assay ID: 10297, Thermo Fisher) were used. Briefly, cells were seeded in 6-well plates at 1 × 10^6^ cells/well for 24 h before transfection. At 90% confluence, the cells were transfected with 30 nmol/L siRNA using Lipofectamine RNAi MAX (# 13778, Invitrogen) in OptiMEM according to the manufacturer’s instructions. Transfection mixes were applied to the cells for 24 h, subsequently removed and replaced with 2 mL of growth media. The cells were cultured for 48 h before use in experiments. The protein expression levels of Piezo1 and Cav-1 were ascertained by Western blot.

### In vitro transwell invasion assay

To assay the effect of compression on cell invasion, standard transwell invasion assay adapted from Bravo-Cordero [62, 63] was performed using 6-well Transwell chambers that were separated as upper and lower chambers by filter membrane with 8 μm pores (# 07–200-169, Corning). For the assay, the transwell filter membrane was coated with 300 μl Matrigel (12 mg/mL, # E1270, Sigma, Burlington, MA) diluted in serum-free DMEM (2 mg/mL final concentration), followed by incubation for 1 h at 37 °C. MDA-MB-231 cells in serum-free medium (5 × 10^5^ cells/well) were placed in the upper chamber, while the lower chamber was filled with 2 mL complete medium. Cells were allowed to grow for 6 h and then compressed for 18 h before being fixed with 4% paraformaldehyde (# 30525–89-4, Electron Microscopy Sciences, Hatfield, PA). The non-invasive cells on the upper chamber were removed with cotton swabs, and the invaded cells in the lower chamber were stained with 0.1% crystal violet (# C6158; Sigma) for 10 min at room temperature, before being examined and imaged by light microscopy at 10X magnification (Olympus BX60; Olympus Corporation, Tokyo, Japan). Then the number of stained cells was counted using ImageJ software (National Institute of Health, Bethesda, MD) and the enhancement of cellular invasion induced by compression was quantified as a percentage (%) of the number of compressed cells over that of the non-compressed cells that had invaded through the filter membrane, i.e. [# of cells in the lower chamber in the presence of a specific weight (experiment group)]/[# of cells in the lower chamber in the absence of a specific weight (control group)]. Results are based on the analysis of 10 random fields per transwell in each condition and each experiment was repeated three times.

### Live fluorescence microscopy

To observe the dynamics of actin, Cav-1, and calcium signaling, live cells expressing Lifeact-RFP, Cav-1-EGFP, and G-GECO were imaged with a spinning disk confocal microscope with a 60X or 100X oil immersion objective (Olympus IX73 with Yokogawa CSU-X1). For live fluorescence microscopy, cells were seeded in a 35 mm glass-bottom dish that was placed in an environmental chamber mounted on the microscope to maintain constant 37 °C, 5% CO_2_, and humidity. Cav-1-EGFP was observed at the excitation wavelength of 488 nm. For dynamic tracking of actin in live cells, the cells were consecutively imaged for up to 60 min, and the images were processed using ImageJ. Cells were observed from both top-down and side view for spatial localization of actin, and caveolae by 3D reconstruction of images in Z-stacks (0.4 μm increments).

### Cell height and nuclear area assay

Cell height and nuclear area can be used to indicate the effect of compression on cells. MDA-MB-231 cells transduced with Lifeact-RFP were plated in glass-bottom dishes at a density of 2 × 10^5^ cells/mL and cultured for 24 h at 37 °C and 5% CO_2_. At 24 h, the cells were incubated with Hoechst 33342 in PBS (1: 2000) for 20 min. 1% agarose disks were UV-treated, incubated in media for 1 h at 37 °C, and then placed on top of the cells. Weights were applied to achieve 200 Pa, 400 Pa, and 600 Pa. For the condition of a control group, an agarose disk was applied without any weight. The agarose disks allow nutrient diffusion and sit in between the weight and the cells. Fluorescence live-cell imaging was performed using a spinning disk confocal microscope. Hoechst and Lifeact-RFP were excited at wavelengths of 405 nm and 561 nm, respectively. Image stacks were taken at 30 min intervals for 2 h. Cell height and nuclear area were quantified by the side-view profiles of Lifeact-RFP images and the top-view profiles of Hoechst 33342 images, respectively, using ImageJ.

### Cell proliferation assay

MDA-MB-231 cells were plated in transwell cell culture inserts at a density of 2 × 10^5^ cells/mL and cultured for 24 h at 37 °C and 5% CO_2_. The cells were then transfected with scramble siRNA or Piezo1 siRNA using Lipofectamine 3000 and cultured for another 24 h. Pre-incubated agarose disks were placed on top of the cells, and weights were applied on top of the agarose disks. After 24 h, the weights and the agarose disks were removed. The media was collected in labeled centrifuge tubes. The cells were detached using 0.05% trypsin, transferred to the corresponding tubes, and spun down at 1000×*g* for 5 min. The cells were then resuspended in 1 mL of fresh media. 50 μL of cell suspension, 55 μL of DMEM, and 5 μL of WST-8 solution were added to each well in a 96-well plate, mixed gently on an orbital shaker, and incubated for 2 h at 37 °C and 5% CO_2_. The absorbance of the samples at a wavelength of 450 nm was measured using a plate reader.

### Cell migration assay

To assay the effect of compression on cell migration, standard wound healing assays were performed using 6-well Transwell chambers that were separated as upper and lower chambers by a filter membrane with 0.4 μm pores (# 07–200-148, Corning). For the assay, MDA-MB-231 cells (1 × 10^6^ cells/well) were placed in the upper chamber, while the lower chamber was filled with 2 mL complete medium. Cells were allowed to grow for 24 h to achieve a confluent monolayer. An experimental wound was made using a sterile micropipette tip, then the cells were washed 3 times with sterile PBS and compressed for 24 h. Wound areas were observed and recorded at 24 h by using a Nikon TiE Perfect Focus System microscope equipped with an 10X objective, an sCMOS camera (Flash 4.0, Hamamatsu Photonics, Japan), and a laser launch controlled by an acousto-optical tunable filter (AOTF). The experimental wound area was quantified manually using “Area measurement” in ImageJ software and normalized to the wound area at the start of the experiment, and the ratio of cell migration was defined by the ratio of the wound healing area of compression-treated groups to that of control groups. Results are based on the analysis of 3 random fields per transwell in each condition and each experiment was repeated three times.

### Evaluation of invadopodia formation and ECM degradation

To determine whether compression enhances cells’ ability to degrade ECM, we examined cells cultured on gelatin substrate for their tendency to form invadopodia and associated gelatin degradation, according to a protocol adapted from Artym et al. [64]. Briefly, glass-bottom dishes were treated with 20% nitric acid for 1 h, washed with H_2_O for 4 times, then incubated with 50 μg/mL poly-L-lysine (# P8920, Sigma) in phosphate buffer solution (PBS) for 15 min and washed with PBS, then further incubated with 0.5% glutaraldehyde in PBS on ice for 15 min followed by thorough washes with PBS. Subsequently, the dishes were coated with 1 mL of gelatin in PBS (1:9 of 0.1% fluorescein isothiocyanate (FITC)-gelatin (# G13186, Invitrogen): 2% porcine gelatin), then washed in PBS, incubated with 5 mg/mL sodium borohydride (NaBH_4_) for 3 min, rinsed in PBS, and then incubated in 10% FBS/DMEM at 37° for 2 h. Afterward, MDA-MB-231 cells were seeded in each dish at 5 × 10^5^ cells per well and incubated for 8 h, and then subjected to compression of either 200 Pa, 400 Pa, or 600 Pa, respectively, for 8 h as aforementioned.

Upon completion of compression, the cells were imaged with live fluorescence microscopy (60X) and the microscopic images were analyzed by using ImageJ to assess the formation of invadopodia and the degradation of gelatin matrix. Invadopodia were defined as F-actin-positive puncta protruding from the cells into the gelatin matrix underneath the cell in our experiments [65]. For each independent experiment that was performed in triplicates, the number of invadopodia per cell was quantified with cells imaged randomly in > 15 microscope view fields, representing a total of ~ 100 cells per experimental condition. At the same time, degradation of the gelatin matrix was quantified as the percentage of the degraded area (dark spots comprised of dense degraded protein products) in the whole area underneath each cell.

### Intracellular Ca^2+^ measurement

To evaluate the intracellular calcium concentration ([Ca^2+^]), we used cells labeled with Fluo-4/AM (# F14201, Thermo Fisher) or transiently expressed with calcium-sensitive reporter G-GECO [66] and then evaluated the intensity of intracellular calcium signaling. For the Fluo-4/AM system, cells were incubated with Fluo-4/AM for 1 h at room temperature (25 ± 2 °C) followed by a 0.5 h wash at 37 °C. For G-GECO systems, cells transfected with G-GECO for 48 h were plated into a glass-bottom dish, which was further incubated for 24 h. Subsequently, the cells were imaged with the spinning disk confocal microscope (60X objective), with fluorescence excitation and emission at 488 nm and 533 nm, respectively. For each experimental group, twenty cells were randomly selected and the fluorescence intensity per cell was quantified using ImageJ.

### Western blot

Western blot assay was used to examine the protein expression and/or activity of Piezo1, Cav-1, Src, and ERK in MDA-MB-231 cells after exposure to control groups or mechanical compression conditions for 4 h. Cells grown on glass-bottom dishes under described assay conditions were lysed using RIPA buffer (# R0278, Sigma) with an added cocktail of protease and phosphatase inhibitors (MS-SAFE, Sigma). The protein concentration of cell lysates was determined using the Protein Assay Reagent (#23227, Thermo Fisher). Cell lysis buffer was combined in 4× SDS sample buffer and 2-mercaptoethanol and incubated at 95 °C for 5 min. After loading an equal amount of protein per lane, SDS-PAGE was performed. The proteins were transferred onto 0.22 μm nitrocellulose membranes (# 66485, Pall Life Sciences) using Pierce G2 Fast Blotter (Thermo Fisher). Following the transfer, the membranes were cut before probing with antibodies to save antibodies. Membranes were first blocked using 5% nonfat milk in 1x TBST (Tris-buffered saline and 0.1% of Tween-20) for 1 h at RT with gentle agitation and incubated with the primary antibodies overnight at 4 °C under mild shaking condition. After washing three times with 1x TBST, membranes were incubated with goat anti-rabbit secondary antibody (DyLight 800, # SA5–10036, Thermo Fisher) or goat anti-mouse secondary antibody (DyLight 680, # 35518, Thermo Fisher) at RT for 1 h. Signals of immunoblots were detected using the Odyssey Infrared Imaging System (LI-COR, Lincoln, NE). Images were cropped to only show the molecular weight regions that are informative for our proteins on interest and were grouped into panels for clearer presentation and easier comprehension. For quantification, the intensity of the gel band was calculated after subtracting the background. The relative protein expression was expressed as a ratio of the band intensity to that of the control group after both of them were normalized to that of GAPDH.

### Immunofluorescence and colocalization analysis

Cells were fixed with 4% paraformaldehyde for 10 min and permeabilized with 0.1% TritonX-100 for 10 min at room temperature. Non-specific sites were blocked using 5% non-fat milk in PBS for 1 h at room temperature. Cells were then incubated in 5% non-fat milk in PBS containing primary antibodies at 1:100 dilution for 1 h at room temperature. After washing with PBS, cells were incubated with Alexa Fluor 594 or 640 conjugated secondary antibody for 60 min at room temperature. Cells were visualized using the spinning disk confocal microscope with a 60X oil immersion objective. For F-actin staining, cells were incubated with 1:100 rhodamine-phalloidin (# PHDR1, Cytoskeleton Inc.) for 60 min at room temperature.

Colocalization of Piezo1 and Cav-1 was analyzed using Fiji software [67] containing a procedure for colocalization analysis, designated as Coloc2, which is based on pixel-intensity-correlation measurements. Pearson coefficient and 2D intensity histograms were recorded to quantify the degree of the colocalization between Piezo1 and Cav-1.

### Statistical analysis

Statistical analysis was done using one-way analysis of variance (ANOVA), followed by post hoc student’s *t* test for multiple comparisons. Statistical significance set to *p < 0.05 and **p < 0.01. All experiments were repeated at least three times and the data expressed as means ± s.e.m. (standard error of the mean).

## Supplementary Information

**Additional file 1: Video S1.**

**Additional file 2: Video S2.**

**Additional file 3.** Supplemental information.

## Data Availability

The datasets supporting the conclusions of this article are included within the article and its additional files.

## Abbreviations

2D: Two dimensional
3D: Three dimensional
[Ca^2+^]: Intracellular calcium concentration
ANOVA: Analysis of variance
Cav-1: Caveolin-1
Ctr: Control
DMEM: Dulbecco’s modified Eagle medium
ECM: Extracellular matrix
EGF: Epidermal growth factor
EGFP: Enhanced green fluorescent protein
EGTA: Ethylene glycol tetraacetic acid
ER: Endoplasmic reticulum
ERK: Extracellular regulated protein kinase
FITC: Isothiocyanate
Gd^3+^: Gadolinium chloride
G-GECO: Green genetically encoded Ca^2+^-indicators for optical imaging
HIF: Hypoxia-inducible factor
KD: Knockdown
MMP: Matrix metalloproteinase
MβCD: Methyl-β-cyclodextrin
qRT-PCR: Quantitative real-time polymerase chain reaction
PBS: Phosphate buffer solution
RFP: Red fluorescent protein
RT: Room temperature
SACs: Stretch-activated ion channels
TRP: Transient receptor potential
WT: Wild type
