# Supplementary material for: Compression enhances invasive phenotype and matrix degradation of breast Cancer cells via Piezo1 activation
Source: BMC Mol Cell Biol. 2022 Jan 3;23:1. doi: 10.1186/s12860-021-00401-6 (PMC8722159; doi:10.1186/s12860-021-00401-6)
Supplement: Supplementary file 3 — Additional file 3. Supplemental information. [file 12860_2021_401_MOESM3_ESM.docx]

**Compression Enhances Invasive Phenotype and Matrix Degradation of Breast Cancer Cells via Piezo1 Activation**

Mingzhi Luo^1,2^, Grace Cai^3^, Kenneth K. Y. Ho^2^, Kang Wen^1^, Zhaowen Tong^3^, Linhong Deng^1,*^, Allen P. Liu^2,3,4,5,6*^

^1^ Institute of Biomedical Engineering and Health Sciences, Changzhou University, Changzhou, Jiangsu, P. R. China

^2^ Department of Mechanical Engineering, University of Michigan, Ann Arbor, Michigan, United States

^3^ Applied Physics Program, University of Michigan, Ann Arbor, Michigan, United States

^4^ Department of Biophysics, University of Michigan, Ann Arbor, Michigan, United States

^5^ Department of Biomedical Engineering, University of Michigan, Ann Arbor, Michigan, United States

^6^ Cellular and Molecular Biology Program, University of Michigan, Ann Arbor, Michigan, United States

* Corresponding author: Linhong Deng, +86-13685207009, dlh@cczu.edu.cn; Allen P. Liu, +1-734-764-7719, [allenliu@umich.edu](mailto:allenliu@umich.edu)


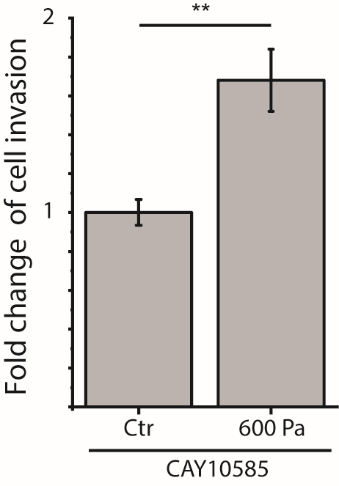


**Figure S1** Compression-enhanced invasion of MDA-MB-231 breast cancer cells did not involve HIF-1α. Quantification of the fold change of invaded cells with HIF-1α inhibitor CAY10585 under the compression (600 Pa) normalized to the control group. Data are presented as means ± s.e.m, n = 4, ^**^ *p* < 0.01 versus Ctr groups. Please refer to Figure 1f for the no-drug condition.


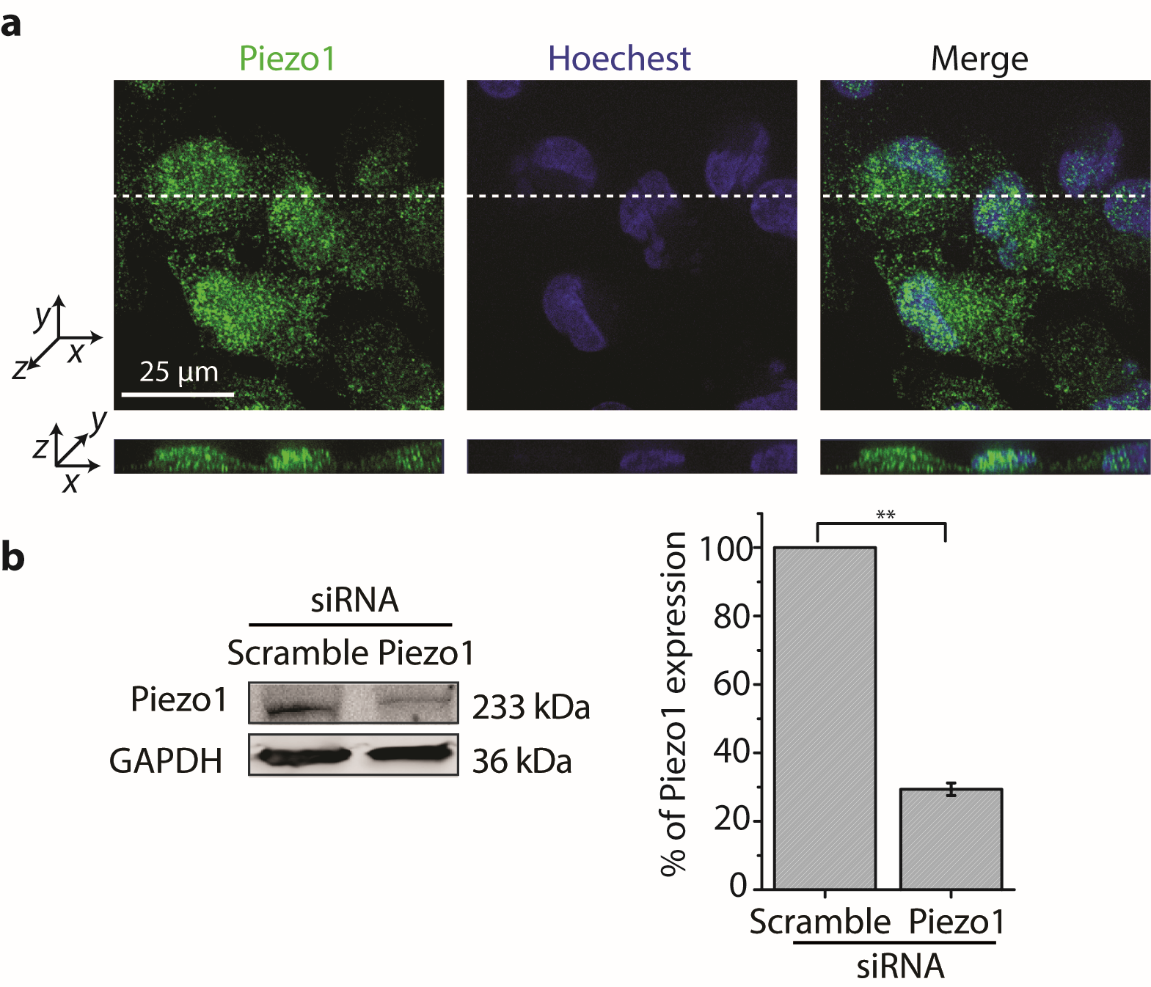


**Figure S2** The distribution and the efficiency of siRNA knockdown (KD) of Piezo1 in MDA-MB-231 breast cancer cells. **a** Representative confocal microscopic images of Piezo1 in MDA-MB-231 cells (100X), Piezo1 (green) and nucleus (blue) were detected with antibodies and Hoechst 33342, respectively, showing puncta structures of Piezo1 localized in the plasma membrane, cytosol, and nucleus. The white dashed line shows the position of the section of *x-z* images. **b** Efficiency of siRNA KD for Piezo1. The cells were transfected with scramble control or siRNA probes for Piezo1 for 48 h and Piezo1 expression was determined by Western blot. Cropped images of Western blots are shown and uncropped images are shown in Fig. S8a. The bar graphs show the percentage of Piezo1 expression in siRNA groups normalized to that at scramble groups (^**^*p* < 0.01 versus Ctr groups, n = 3).

**
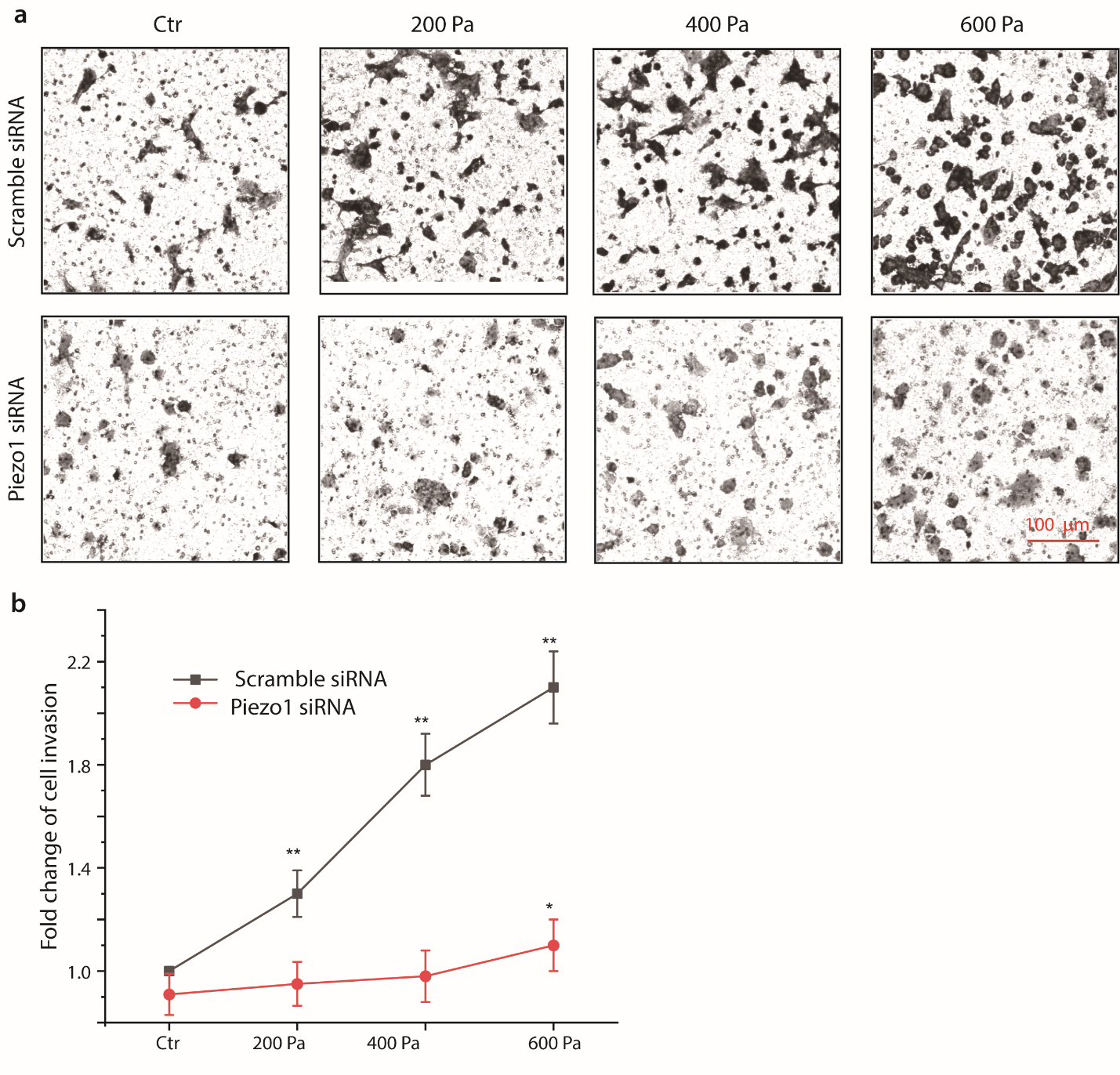
**

**Figure S3** The role of Piezo1 in compression-enhanced invasion of 4T1 breast cancer cells. **a** Representative images of invaded cells stained with crystal violet under different compression. Bar = 100 μm. **b** Quantification of fold change of invaded cells to the control (Ctr) group pretreated with scramble or Piezo1 siRNA. Data were presented as means ± s.e.m, n = 3. ^*^ *p* < 0.05 versus Ctr groups; ^**^*p* < 0.01 versus Ctr groups.

**
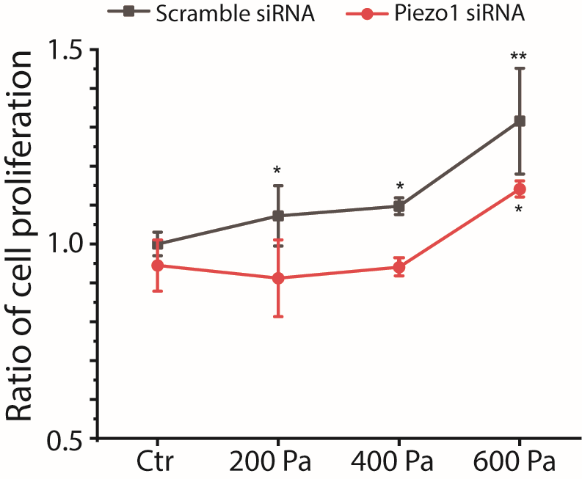
**

**Figure S4** Cell proliferation of MDA-MB-231 with or without Piezo1 siRNA treatment as a function of compression normalized to cell proliferation at control (Ctr) groups. Data are presented as means ± s.e.m., n = 3, ^*^ *p* < 0.05 versus Ctr groups and ^**^ *p* < 0.01 versus Ctr groups.

**
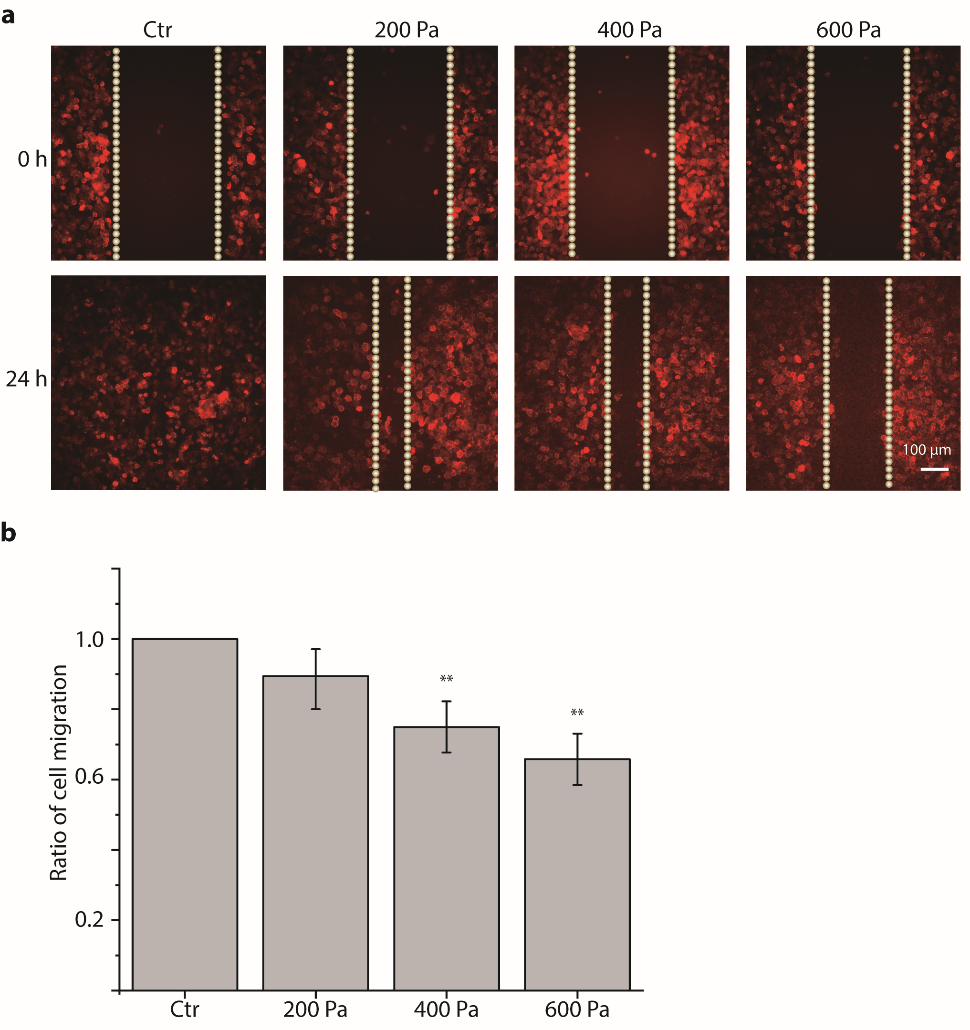
**

**Figure S5** The effect of compression for 24 h on the 2D cell migration of MDA-MB-231 breast cancer cells evaluated with wound healing assay in a transwell experiment setup. **a** Representative images of cells expressing Lifeact-RFP under the absence or presence of compression at 200, 400, and 600 Pa. Bar = 100 μm. **b** Quantification of the ratio of cell migration to the control (Ctr) group with 1% agarose. Data are presented as means ± s.e.m, n = 3, ^**^ *p* < 0.01 versus Ctr groups.

**
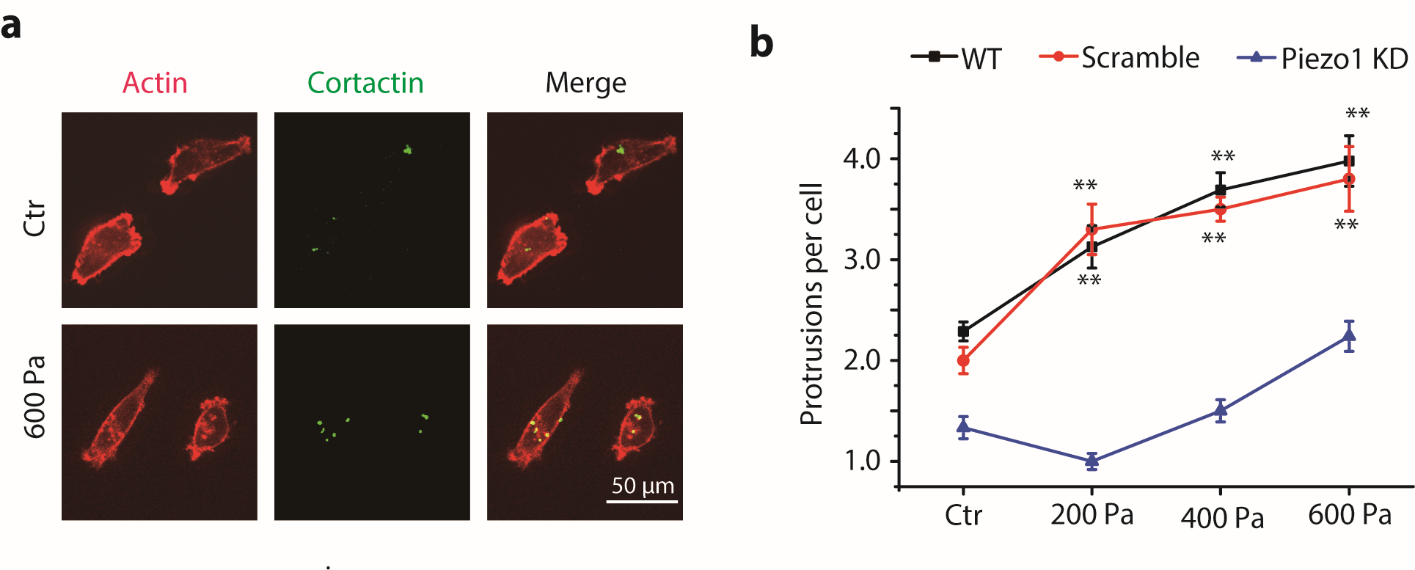
 Figure S6** Actin protrusions in MDA-MB-231 cells. **a** Representative images (red: actin, green: cortactin) of protrusions in MDA-MB-231 cells without (upper panel, control, Ctr) or with (bottom panel) 600 Pa compression. Control and compression-treated MDA-MB-231 cells were cultured on a gelatin-coated glass-bottom dish for 8 h, then fixed and stained with phalloidin to visualize F-actin (red) and antibody for cortactin (green) to identify the invadopodia. **b** The number of protrusions per cell under compression with or without Piezo1 KD. F-actin-positive puncta inside cells with certain size (diameter (i.e., pixel numbers) > 6) were counted as invadopodia. Data are presented as ± s.e.m, n = 20-30, ^**^ *p* < 0.01 versus Ctr groups.

**
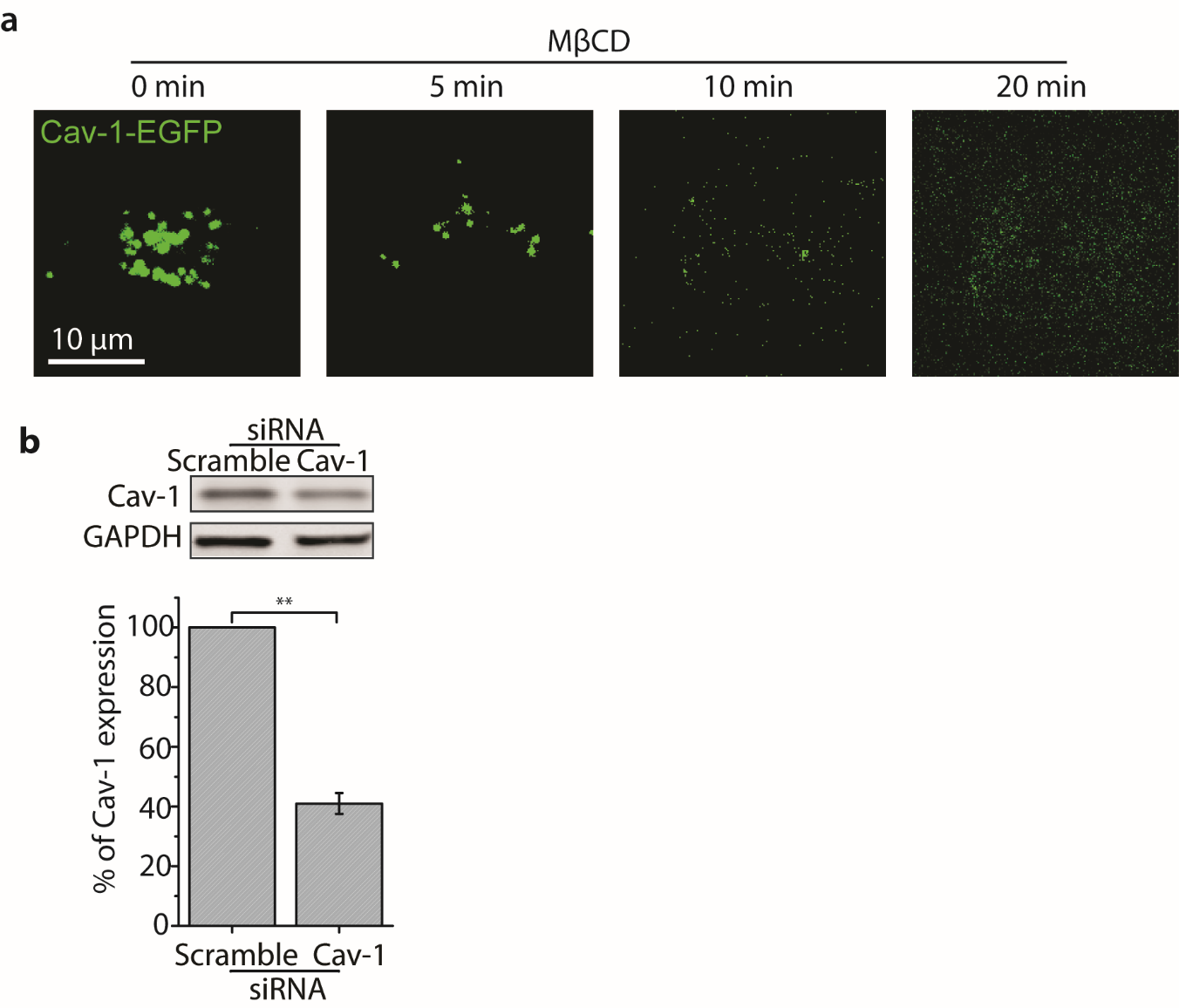
**

**Figure S7** Caveolae regulate the function of Piezo1 in responding to compression in MDA-MB-231 cells. **a** Effect of methyl-beta-cyclodextrin (MβCD) on removing caveolae from the plasma membrane. Cells expressing Cav-1-EGFP were treated with 5 mM of MβCD for 5 min, 10 min, and 20 min at 37 °C and imaged with a confocal microscope (100X, bar = 10 μm). **b** Efficiency of siRNA knockdown (KD) for caveolin1 (Cav-1). Cropped images of Western blots are shown and uncropped images are shown in Fig. S8c. The bar graphs show the percentage of Piezo1 in scramble and Cav-1 siRNA groups (^**^ *p* <0.01 versus scramble groups, n = 3).

**
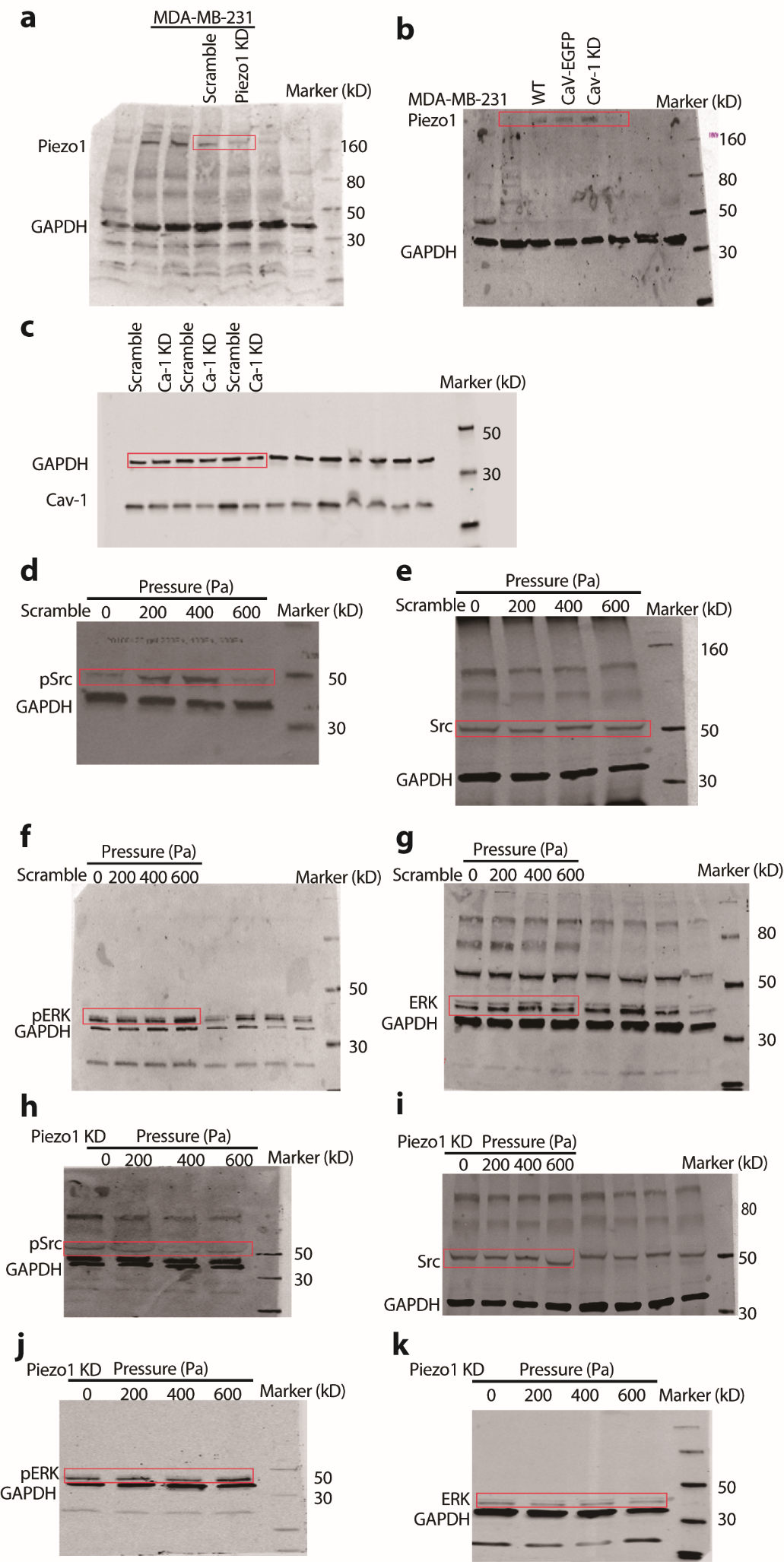
**

**Figure S8** Full-size images of Western blots. **a** Full-size image of Western blots to evaluate the efficiency of siRNA KD for Piezo1 shown in Figure S2b. **b** Full-size image of Western blots to evaluate Piezo1 expression in wild type (WT), Cav-1 EGFP expressing, and Cav-1 KD MDA-MB-231 cells shown in Figure 4c. **c** Full-size image of Western blots to evaluate the efficiency of siRNA knockdown (KD) for caveolin1 (Cav-1) in MDA-MB-231 cells.  **d**, **e**, **f**, **g** are full-size images of Western blots for Src, p-Src, ERK, p-ERK in MDA-MB-231 cells pretreated with scramble probes in the absence or presence of compression at 200, 400, 600 Pa shown in Figure 5a, respectively. **h**, **i**, **j**, **k** are full-size images of Western blots for Src, p-Src, ERK, p-ERK in MDA-MB-231 cells pretreated with siRNA for Piezo1 in the absence or presence of compression at 200, 400, 600 Pa shown in Figure 5b, respectively. The red boxes indicate the cropped regions.

**Supplementary video 1** Representative video recording the real-time intracellular [Ca^2+^] response of MDA-MB-231 labeled with Fluo-4/AM. The Fluo-4/AM loaded cells were first recorded for 1 min, which was used for the calculation of baseline calcium signaling. At the end of the first 1 min (60 s), 600 Pa compression was applied to the cells, and the recording of the calcium signaling in the cells was kept for another 1 min.

**Supplementary video 2** Representative video recording the real-time intracellular [Ca^2+^] response of MDA-MB-231 transiently expressing G-GECO**.** The G-GECO loaded cells were first recorded for 1 min, which was used for the calculation of baseline calcium signaling. At the end of the first 1 min (60 s), 600 Pa compression was applied to the cells, and the recording of the calcium signaling in the cells was kept for another 9 min.
